# Vaccines elicit highly conserved cellular immunity to SARS-CoV-2 Omicron

**DOI:** 10.1038/s41586-022-04465-y

**Published:** 2022-01-31

**Authors:** Jinyan Liu, Abishek Chandrashekar, Daniel Sellers, Julia Barrett, Catherine Jacob-Dolan, Michelle Lifton, Katherine McMahan, Michaela Sciacca, Haley VanWyk, Cindy Wu, Jingyou Yu, Ai-ris Y. Collier, Dan H. Barouch

**Affiliations:** 1 Beth Israel Deaconess Medical Center, Boston, MA USA; 2 Ragon Institute of MGH, MIT, and Harvard, Cambridge, MA USA

**Keywords:** Vaccines, T cells, Vaccines, SARS-CoV-2

## Abstract

The highly mutated SARS-CoV-2 Omicron (B.1.1.529) variant has been shown to evade a substantial fraction of neutralizing antibody responses elicited by current vaccines that encode the WA1/2020 spike protein^1^. Cellular immune responses, particularly CD8^+^ T cell responses, probably contribute to protection against severe SARS-CoV-2 infection^2–6^. Here we show that cellular immunity induced by current vaccines against SARS-CoV-2 is highly conserved to the SARS-CoV-2 Omicron spike protein. Individuals who received the Ad26.COV2.S or BNT162b2 vaccines demonstrated durable spike-specific CD8^+^ and CD4^+^ T cell responses, which showed extensive cross-reactivity against both the Delta and the Omicron variants, including in central and effector memory cellular subpopulations. Median Omicron spike-specific CD8^+^ T cell responses were 82–84% of the WA1/2020 spike-specific CD8^+^ T cell responses. These data provide immunological context for the observation that current vaccines still show robust protection against severe disease with the SARS-CoV-2 Omicron variant despite the substantially reduced neutralizing antibody responses^7,8^.

## Abstract (web summary)

Current vaccines induce broadly cross-reactive cellular immunity against SARS-CoV-2 variants, including Omicron, and provide protection against severe disease despite a substantially reduced neutralizing antibody response.

## Main

Recent studies have shown that vaccine-elicited neutralizing antibodies (NAbs) are substantially reduced to the highly mutated SARS-CoV-2 Omicron variant^1^. To evaluate the cross-reactivity of vaccine-elicited cellular immune responses against the SARS-CoV-2 Omicron variant, we assessed CD8^+^ and CD4^+^ T cell responses in 47 individuals who were vaccinated with the adenovirus vector-based Ad26.COV2.S vaccine^9^ (Johnson & Johnson; *n* = 20) or the mRNA-based BNT162b2 vaccine^10^ (Pfizer; *n* = 27) in Boston, MA, USA (Extended Data Table 1).

## Humoral immune responses

**Fig. 1:**
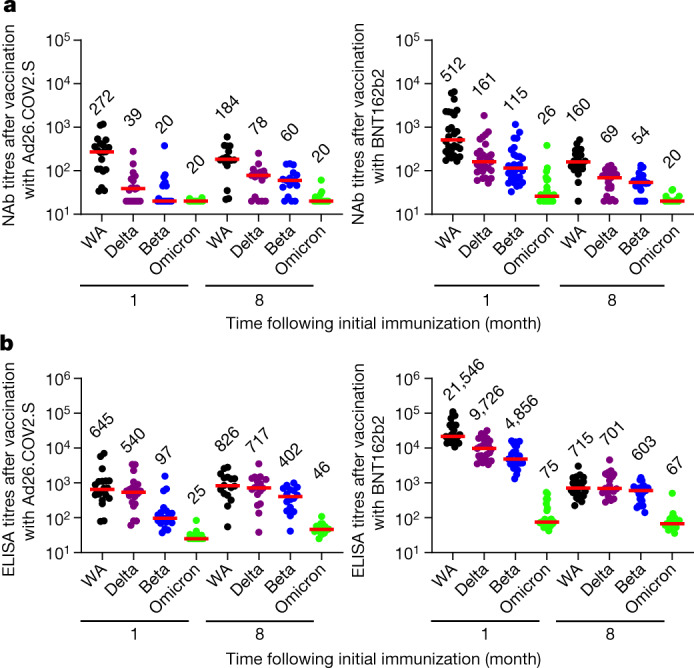
Humoral immune responses to Omicron. Antibody responses at months 1 and 8 following final vaccination with Ad26.COV2.S (*n* = 20) or BNT162b2 (*n* = 27). **a**, Neutralizing antibody (NAb) titres by a luciferase-based pseudovirus neutralization assay. **b**, Receptor-binding domain (RBD)-specific binding antibody titres by ELISA. Responses were measured against the SARS-CoV-2 WA1/2020 (WA), B.1.617.2 (Delta), B.1.351 (Beta) and B.1.1.529 (Omicron) variants. Medians (red bars) are depicted and numerically shown.

**Extended Data Fig. 1:**
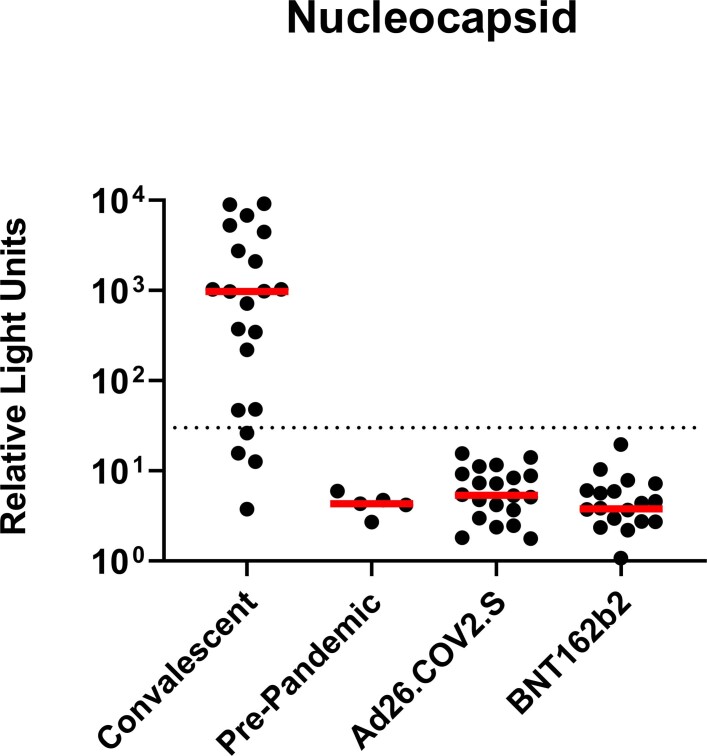
Nucleocapsid antibody responses. Nucleocapsid antibody responses at month 8 following final vaccination with Ad26.COV2.S (N = 20) or BNT162b2 (N = 27) by meso-scale discovery (MSD) electrochemoluminscent assay. SARS-CoV-2 convalescent and pre-pandemic samples were included as positive and negative controls, respectively. Relative light units are shown.

**Extended Data Fig. 2:**
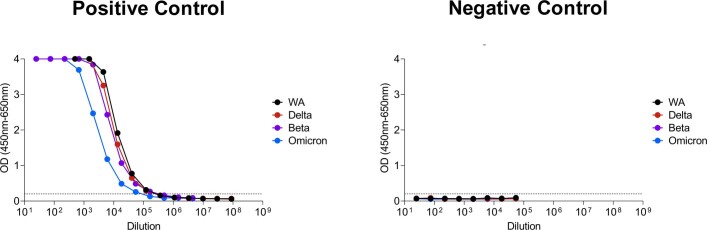
ELISA reactivity against WA1/2020, Beta, Delta, and Omicron RBD proteins. Positive and negative control standards were assessed by ELISA against WA1/2020, Beta, Delta, and Omicron RBD proteins. The positive control standards were known to have 2-3 fold lower antibody titers to Omicron.

All individuals were SARS-CoV-2 naive by history and also had negative antibody responses to nucleocapsid (Extended Data Fig. 1). Following vaccination with BNT162b2, we observed high WA1/2020-specific pseudovirus NAb responses at month 1, followed by a sharp decline by month 8 (*P* < 0.0001, two-tailed Mann–Whitney test), as expected^11,12^ (Fig. 1a). Following vaccination with Ad26.COV2.S, there were initial substantially lower WA1/2020-specific pseudovirus NAb responses at month 1, but these responses were more durable and persisted at month 8 (refs. ^11,13^) (Fig. 1a). However, minimal cross-reactive Omicron-specific NAbs were observed for both vaccines (*P* < 0.0001 for both, two-tailed Mann–Whitney tests) (Fig. 1a), consistent with recent data in the absence of additional boosting^1^. The responses of receptor-binding domain-specific binding antibodies were assessed by ELISA and showed similar trends, with minimal cross-reactive Omicron receptor-binding domain-specific binding antibodies (Fig. 1b, Extended Data Fig. 2).

## Cellular immune responses

**Fig. 2:**
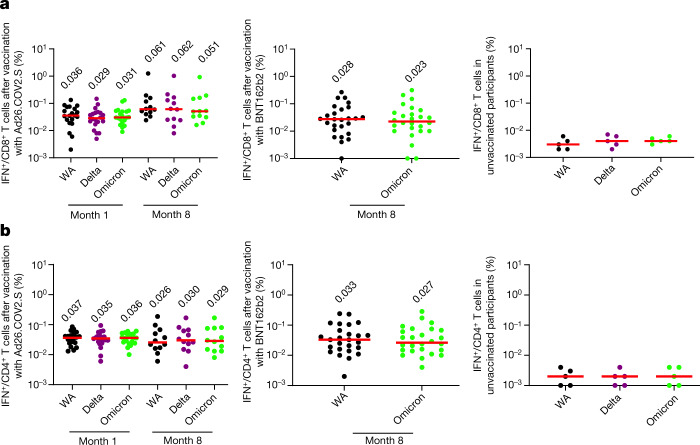
Cellular immune responses to Omicron. T cell responses at months 1 and 8 following final vaccination with Ad26.COV2.S (*n* = 20) or BNT162b2 (*n* = 27). **a**, **b**, Pooled peptide spike-specific IFNγ CD8^+^ T cell responses (**a**) and CD4^+^ T cell responses (**b**) by intracellular cytokine staining assays. Responses were measured against the SARS-CoV-2 WA1/2020, B.1.617.2 (Delta) and B.1.1.529 (Omicron) variants. Responses in five unvaccinated, uninfected individuals are also shown. Media backgrounds were subtracted from the specific values. Medians (red bars) are depicted and numerically shown.

**Fig. 3:**
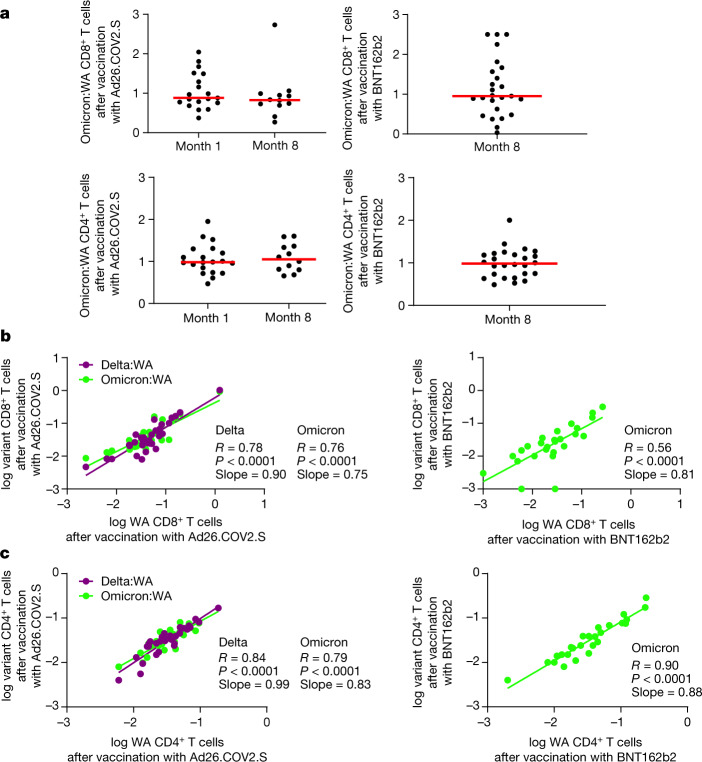
Correlations of variant-specific and WA1/2020-specific cellular immune responses. **a**, Ratio of Omicron to WA1/2020 CD8^+^ (top) and CD4^+^ (bottom) T cell responses in individual participants. **b**, **c**, Correlations of log Delta-specific and Omicron-specific to log WA1/2020-specific CD8^+^ T cell responses (**b**) and CD4^+^ T cell responses (**c**) by intracellular cytokine staining assays. Two-sided unadjusted *P* and *R* values for linear regression correlations are shown, and lines of best fit and slopes are depicted.

**Extended Data Fig. 3:**
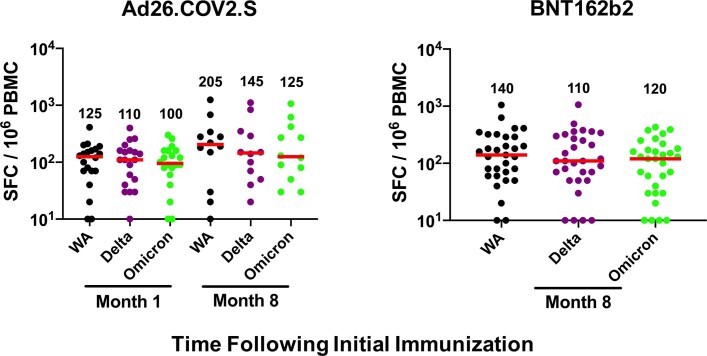
Cellular immune responses to Omicron by ELISPOT assays. Spike-specific IFN-γ ELISPOT assays at month 1 and 8 following final vaccination with Ad26.COV2.S (N = 20) or BNT162b2 (N = 27). Responses were measured against the SARS-CoV-2 WA1/2020, B.1.617.2 (Delta), and B.1.1.529 (Omicron) variants. Medians (red bars) are depicted and numerically shown.

**Extended Data Fig. 4:**
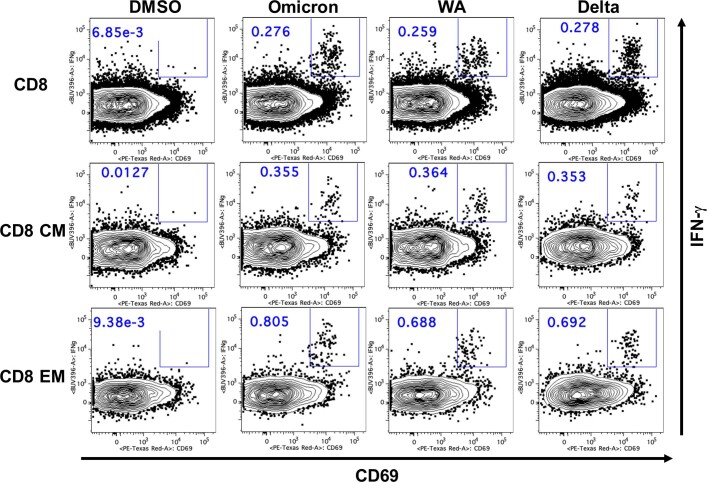
Representative CD8+ T cell responses by flow cytometry. Representative of 47 samples is shown.

**Extended Data Fig. 5:**
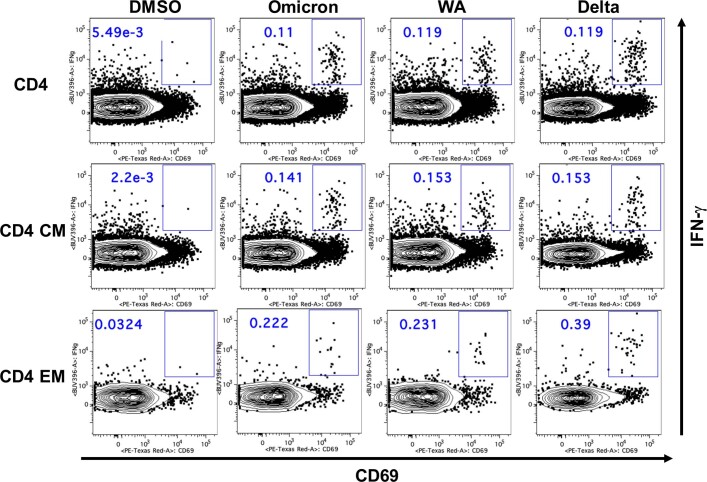
Representative CD4+ T cell responses by flow cytometry. Representative of 47 samples is shown.

**Extended Data Fig. 6:**
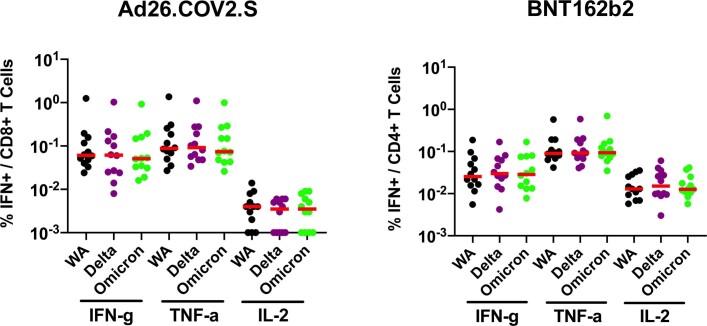
Cellular immune responses to Omicron by intracellular cytokine staining assays. Spike-specific IFN-γ, TNF-α, and IL-2 CD8+ and CD4+ T cell responses by intracellular cytokine staining assays at month 8 following final vaccination with Ad26.COV2.S (N = 20). Responses were measured against the SARS-CoV-2 WA1/2020, B.1.617.2 (Delta), and B.1.1.529 (Omicron) variants. Medians (red bars) are depicted and numerically shown.

In contrast to antibody responses, spike-specific cellular immune responses assessed by pooled peptide IFNγ ELISPOT assays showed substantial cross-reactivity to Omicron (Extended Data Fig. 3, Supplementary Table 1). We next assessed spike-specific CD8^+^ and CD4^+^ T cell responses by intracellular cytokine staining assays (Extended Data Figs. 4, 5, Supplementary Table 1). Ad26.COV2.S induced median spike-specific IFNγ CD8^+^ T cell responses of 0.061%, 0.062% and 0.051% against WA1/2020, Delta and Omicron, respectively, at month 8 following vaccination (Fig. 2a). BNT162b2 induced median spike-specific IFNγ CD8^+^ T cell responses of 0.028% and 0.023% against WA1/2020 and Omicron, respectively, at month 8 following vaccination (Fig. 2a). These data suggest that median Omicron-specific CD8^+^ T cell responses were 82–84% cross-reactive with WA1/2020-specific CD8^+^ T cell responses (the *P* value was not significant; two-tailed Mann–Whitney test). Spike-specific IFNγ CD4^+^ T cell responses elicited by Ad26.COV2.S were a median of 0.026%, 0.030% and 0.029% against WA1/2020, Delta and Omicron, respectively, and by BNT162b2 were a median of 0.033% and 0.027% against WA1/2020 and Omicron, respectively, at month 8, indicating that median Omicron-specific CD4^+^ T cell responses were 82–100% cross-reactive with WA1/2020-specific CD4^+^ T cell responses (the *P* value was not significant; two-tailed Mann–Whitney test) (Fig. 2b). These data demonstrate substantial CD8^+^ and CD4^+^ T cell cross-reactivity to Omicron, although responses may be impacted more in select individuals (Fig. 3a). Substantial Omicron cross-reactivity was also observed for spike-specific IFNγ-secreting, TNF-secreting and IL-2-secreting CD8^+^ and CD4^+^ T cell responses (Extended Data Fig. 6). By contrast, unvaccinated, uninfected individuals had negligible spike-specific CD8^+^ and CD4^+^ T cell responses (Fig. 2a, b).

Omicron-specific CD8^+^ T cell responses correlated with WA1/2020-specific CD8^+^ T cell responses for the Ad26.COV2.S vaccine for both timepoints (*R* = 0.78, *P* < 0.0001, slope = 0.75) and the BNT162b2 vaccine (*R* = 0.56, *P* < 0.0001, slope = 0.81), although two individuals had undetectable Omicron-specific CD8^+^ T cell responses following vaccination with BNT162b2 (Fig. 3b). Similarly, Omicron-specific CD4^+^ T cell responses correlated with WA1/2020-specific CD4^+^ T cell responses for both the Ad26.COV2.S vaccine (*R* = 0.79, *P* < 0.0001, slope = 0.83) and the BNT162b2 vaccine (*R* = 0.90, *P* < 0.0001, slope = 0.88) (Fig. 3c).

**Fig. 4:**
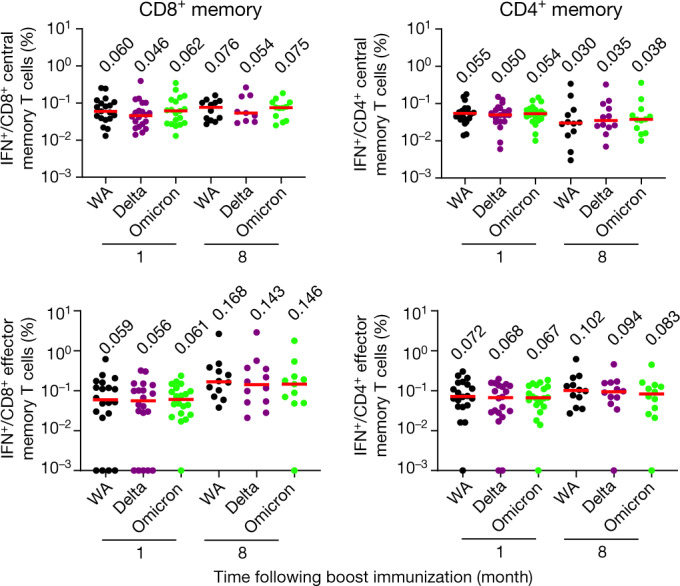
Cellular immune memory subpopulations to Omicron. Pooled peptide spike-specific IFNγ CD8^+^ and CD4^+^ central memory (CD45RA^−^CD27^+^) and effector memory (CD45RA^−^CD27^−^) T cell responses by intracellular cytokine staining assays at months 1 and 8 following final vaccination with Ad26.COV2.S (*n* = 20). Responses were measured against the SARS-CoV-2 WA1/2020, B.1.617.2 (Delta) and B.1.1.529 (Omicron) variants. Medians (red bars) are depicted and numerically shown.

Spike-specific IFNγ CD8^+^ and CD4^+^ T cell central (CD45RA^−^CD27^+^) and effector (CD45RA^−^CD27^−^) memory subpopulations elicited by Ad26.COV2.S also showed extensive cross-reactivity to Delta and Omicron variants. At month 8, CD8^+^ T cell central memory responses were 0.076%, 0.054% and 0.075%, CD8^+^ T cell effector memory responses were 0.168%, 0.143% and 0.146%, CD4^+^ T cell central memory responses were 0.030%, 0.035% and 0.038%, and CD4^+^ T cell effector memory responses were 0.102%, 0.094% and 0.083% against WA1/202, Delta and Omicron, respectively (Fig. 4).

## Discussion

Our data demonstrate that Ad26.COV2.S and BNT162b2 elicit broadly cross-reactive cellular immunity against SARS-CoV-2 variants including Omicron. The consistency of these observations across two different vaccine platform technologies (viral vector and mRNA) suggests the generalizability of these findings. The extensive cross-reactivity of Omicron-specific CD8^+^ and CD4^+^ T cell responses contrasts sharply with the marked reduction of Omicron-specific antibody responses. These data are consistent with previous studies that have shown greater cross-reactivity of vaccine-elicited cellular immune responses than humoral immune responses against the SARS-CoV-2 Alpha, Beta and Gamma variants^14^. T cell responses target multiple regions in the spike protein, consistent with the largely preserved cellular immune responses to Omicron^6,14^. The 82–84% cross-reactivity of CD8^+^ T cell responses to Omicron is also consistent with theoretical predictions based on the Omicron mutations. Limitations of our study include the use of high doses of peptides with costimulation in the intracellular cytokine staining assays, and the lack of assessing the effect of mutations on antigen processing.

Preclinical studies have shown that CD8^+^ T cells contribute to protection against SARS-CoV-2 in rhesus macaques, particularly when antibody responses are suboptimal^5^. Durable CD8^+^ and CD4^+^ T cell responses have also been reported following infection and vaccination^2–4,6,11,13,15,16^. Given the role of CD8^+^ T cells in clearance of viral infections, it is likely that cellular immunity contributes substantially to vaccine protection against severe SARS-CoV-2 disease. This may be particularly relevant for Omicron, which dramatically evades neutralizing antibody responses. Recent studies have shown that Ad26.COV2.S and BNT162b2 provided 85% and 70% protection, respectively, against hospitalization due to the Omicron variant in South Africa^7,8^. Our data provide immunological context for the observation that current vaccines still provide robust protection against severe disease due to the SARS-CoV-2 Omicron variant despite substantially reduced neutralizing antibody responses.

## Methods

### Study population

Samples from individuals who received the BNT162b2 vaccine were obtained from the Beth Israel Deaconess Medical Center (BIDMC) specimen biorepository. Samples from individuals who received Ad26.COV2.S were obtained from the COV1001 study (NCT04436276). Both studies were approved by the BIDMC Institutional Review Board. All participants provided informed consent. Individuals were excluded from this study if they had a history of SARS-CoV-2 infection, received other COVID-19 vaccines or received immunosuppressive medications.

### Pseudovirus NAb assay

The SARS-CoV-2 pseudoviruses expressing a luciferase reporter gene were used to measure pseudovirus NAbs. In brief, the packaging construct psPAX2 (AIDS Resource and Reagent Program), the luciferase reporter plasmid pLenti-CMV Puro-Luc (Addgene) and the spike protein expressing pcDNA3.1-SARS-CoV-2 SΔCT were co-transfected into HEK293T cells (American Type Culture Collection (ATCC) CRL_3216) with Lipofectamine 2000 (Thermo Fisher Scientific). Pseudoviruses of SARS-CoV-2 variants were generated by using the WA1/2020 strain (Wuhan/WIV04/2019, GISAID accession ID: EPI_ISL_402124), the B.1.1.7 variant (Alpha, GISAID accession ID: EPI_ISL_601443), the B.1.351 variant (Beta, GISAID accession ID: EPI_ISL_712096), the B.1.617.2 variant (Delta, GISAID accession ID: EPI_ISL_2020950) or the B.1.1.529 variant (Omicron, GISAID ID: EPI_ISL_7358094.2). The supernatants containing the pseudotype viruses were collected 48 h after transfection; pseudotype viruses were purified by filtration with a 0.45-μm filter. To determine the neutralization activity of human serum, HEK293T-hACE2 cells were seeded in 96-well tissue culture plates at a density of 1.75 × 10^4^ cells per well overnight. Threefold serial dilutions of heat-inactivated serum samples were prepared and mixed with 50 μl of pseudovirus. The mixturewas incubated at 37 °C for 1 h before adding to HEK293T-hACE2 cells. After 48 h, cells were lysed in a Steady-Glo Luciferase Assay (Promega) according to the manufacturer’s instructions. SARS-CoV-2 neutralization titres were defined as the sample dilution at which a 50% reduction (NT50) in relative light units was observed relative to the average of the virus control wells.

### ELISA

SARS-CoV-2 spike receptor-binding domain (RBD)-specific binding antibodies in serum were assessed by ELISA. Ninety-six-well plates were coated with 2 μg ml^−1^ of similarly produced SARS-CoV-2 WA1/2020, B.1.617.2 (Delta), B.1.351 (Beta) or B.1.1.529 (Omicron) RBD protein in 1× Dulbecco’s phosphate-buffered saline (DPBS) and incubated at 4 °C overnight. Assay performance was similar for these four RBD proteins. After incubation, plates were washed once with wash buffer (0.05% Tween 20 in 1× DPBS) and blocked with 350 μl of casein block solution per well for 2–3 h at room temperature. Following incubation, block solution was discarded and plates were blotted dry. Serial dilutions of heat-inactivated serum diluted in casein block were added to wells, and plates were incubated for 1 h at room temperature, before three more washes and a 1-h incubation with a 1:4,000 dilution of anti-human IgG horseradish peroxidase (Invitrogen, Thermo Fisher Scientific) at room temperature in the dark. Plates were washed three times, and 100 μl of SeraCare KPL TMB SureBlue Start solution was added to each well; plate development was halted by adding 100 μl of SeraCare KPL TMB Stop solution per well. The absorbance at 450 nm, with a reference at 650 nm, was recorded with a VersaMax microplate reader (Molecular Devices). For each sample, the ELISA end point titre was calculated using a four-parameter logistic curve fit to calculate the reciprocal serum dilution that yields a corrected absorbance value (450–650 nm) of 0.2. Interpolated end point titres were reported.

### Enzyme-linked immunospot assay

Peptide pools were 16 amino acid peptides overlapping by 11 amino acids spanning the SARS-CoV-2 WA1/2020, B.1.617.2 (Delta) or B.1.1.529 (Omicron; GISAID ID: EPI_ISL_7358094.2) spike proteins (21st Century Biochemicals). Enzyme-linked immunospot (ELISPOT) plates were coated with mouse anti-human IFNγ monoclonal antibody from MabTech at 1 µg per well and incubated overnight at 4 °C. Plates were washed with DPBS and blocked with R10 media (RPMI with 10% heat-inactivated FBS with 1% of 100× penicillin–streptomycin, 1 M HEPES, 100 mM sodium pyruvate, 200 mM l-glutamine and 0.1% of 55 mM 2-mercaptoethanol) for 2–4 h at 37 °C. SARS-CoV-2 pooled S peptides from SARS-CoV-2 WA1/2020, B.1.617.2 (Delta) or B.1.1.529 (Omicron) (21st Century Biochemicals) were prepared and plated at a concentration of 2 µg per well, and 100,000 cells per well were added to the plate. The peptides and cells were incubated for 15–20 h at 37 °C. All steps following this incubation were performed at room temperature. The plates were washed with ELISPOT wash buffer and incubated for 2–4 h with biotinylated mouse anti-human IFNγ monoclonal antibody from MabTech (1 µg ml^−1^). The plates were washed a second time and incubated for 2–3 h with conjugated goat anti-biotin AP from Rockland, Inc. (1.33 µg ml^−1^). The final wash was followed by the addition of nitro-blue tetrazolium chloride/5-bromo-4-chloro 3 ‘indolyphosphate p-toludine salt (NBT/BCIP chromagen) substrate solution for 7 min. The chromagen was discarded and the plates were washed with water and dried in a dim place for 24 h. Plates were scanned and counted on a Cellular Technologies Limited Immunospot Analyzer.

### Intracellular cytokine staining assay

CD4^+^ and CD8^+^ T cell responses were quantitated by pooled peptide-stimulated intracellular cytokine staining (ICS) assays. Peptide pools were 16 amino acid peptides overlapping by 11 amino acids spanning the SARS-CoV-2 WA1/2020, B.1.617.2 (Delta) or B.1.1.529 (Omicron; GISAID ID: EPI_ISL_7358094.2) spike proteins (21st Century Biochemicals). 10^6^ peripheral blood mononuclear cells were resuspended in 100 µl of R10 media supplemented with CD49d monoclonal antibody (1 µg ml^−1^) and CD28 monoclonal antibody (1 µg ml^−1^). Each sample was assessed with mock (100 µl of R10 plus 0.5% DMSO; background control), peptides (2 µg ml^−1^) and/or 10 pg ml^−1^ phorbol myristate acetate (PMA) and 1 µg ml^−1^ ionomycin (Sigma-Aldrich) (100 µl; positive control) and incubated at 37 °C for 1 h. After incubation, 0.25 µl of GolgiStop (BD) and 0.25 µl of GolgiPlug (BD) in 50 µl of R10 was added to each well and incubated at 37 °C for 8 h and then held at 4 °C overnight. The next day, the cells were washed twice with DPBS, stained with aqua live/dead dye for 10 min and then stained with predetermined titres of monoclonal antibodies ti CD279 (clone EH12.1, BB700), CD4 (clone L200, BV711), CD27 (clone M-T271, BUV563), CD8 (clone SK1, BUV805) and CD45RA (clone 5H9, APC H7) for 30 min. Cells were then washed twice with 2% FBS/DPBS buffer and incubated for 15 min with 200 µl of BD CytoFix/CytoPerm Fixation/Permeabilization solution. Cells were washed twice with 1X Perm Wash buffer (BD Perm/Wash Buffer 10X in the CytoFix/CytoPerm Fixation/ Permeabilization kit diluted with MilliQ water and passed through a 0.22-µm filter) and stained intracellularly with monoclonal antibodies to Ki67 (clone B56, BB515), IL-21 (clone 3A3-N2.1, PE), CD69 (clone TP1.55.3, ECD), IL-10 (clone JES3-9D7, PE CY7), IL-13 (clone JES10-5A2, BV421), IL-4 (clone MP4-25D2, BV605), TNF (clone Mab11, BV650), IL-17 (clone N49-653, BV750), IFNγ (clone B27, BUV395), IL-2 (clone MQ1-17H12, BUV737), IL-6 (clone MQ2-13A5, APC) and CD3 (clone SP34.2, Alexa 700) for 30 min. Cells were washed twice with 1X Perm Wash buffer and fixed with 250 µl of freshly prepared 1.5% formaldehyde. Fixed cells were transferred to a 96-well round bottom plate and analysed by BD FACSymphony system. Data were analysed using FlowJo v9.9.

### Statistical analysis

Descriptive statistics and logistic regression were performed using GraphPad Prism 8.4.3, (GraphPad Software). Immunological data were generated in duplicate and were compared by Mann–Whitney tests. Correlations were evaluated by linear regression. *P* < 0.05 were considered significant.

### Reporting summary

Further information on research design is available in the Nature Research Reporting Summary linked to this paper.

## Online content

Any methods, additional references, Nature Research reporting summaries, source data, extended data, supplementary information, acknowledgements, peer review information; details of author contributions and competing interests; and statements of data and code availability are available at 10.1038/s41586-022-04465-y.

### Supplementary information

Reporting Summary

Supplementary Table 1 Peptide sequences for WA1/2020 and Omicron spike.

## Figures and Tables

**Extended Data Table 1:**
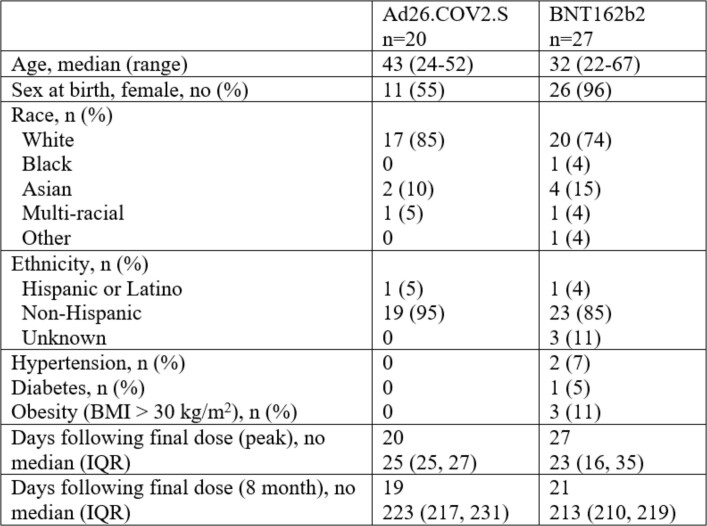
Characteristics of study population

BMI, body mass index. IQR, interquartile range.

## Data Availability

All data are available in the paper or the supplementary material.
