# Supplementary material for: Vaccines elicit highly conserved cellular immunity to SARS-CoV-2 Omicron
Source: Nature. 2022 Jan 31;603(7901):493–6. doi: 10.1038/s41586-022-04465-y (PMC8930761; doi:10.1038/s41586-022-04465-y)
Supplement: Supplementary file 1 — Reporting Summary [file 41586_2022_4465_MOESM1_ESM.pdf]

nature portfolio

Corresponding author(s): Dan Barouch

Last updated by author(s): Jan 19, 2022

Reporting Summary

Nature Portfolio wishes to improve the reproducibility of the work that we publish. This form provides structure for consistency and transparency in reporting. For further information on Nature Portfolio policies, see our Editorial Policies and the Editorial Policy Checklist.

Statistics

For all statistical analyses, confirm that the following items are present in the figure legend, table legend, main text, or Methods section.

n/a	Confirmed	
☐	☒	The exact sample size (*n*) for each experimental group/condition, given as a discrete number and unit of measurement
☐	☒	A statement on whether measurements were taken from distinct samples or whether the same sample was measured repeatedly
☐	☒	The statistical test(s) used AND whether they are one- or two-sided<br>*Only common tests should be described solely by name; describe more complex techniques in the Methods section.*
☒	☐	A description of all covariates tested
☐	☒	A description of any assumptions or corrections, such as tests of normality and adjustment for multiple comparisons
☐	☒	A full description of the statistical parameters including central tendency (e.g. means) or other basic estimates (e.g. regression coefficient) AND variation (e.g. standard deviation) or associated estimates of uncertainty (e.g. confidence intervals)
☐	☒	For null hypothesis testing, the test statistic (e.g. *F*, *t*, *r*) with confidence intervals, effect sizes, degrees of freedom and *P* value noted<br>*Give P values as exact values whenever suitable.*
☒	☐	For Bayesian analysis, information on the choice of priors and Markov chain Monte Carlo settings
☒	☐	For hierarchical and complex designs, identification of the appropriate level for tests and full reporting of outcomes
☒	☐	Estimates of effect sizes (e.g. Cohen's *d*, Pearson's *r*), indicating how they were calculated

Our web collection on statistics for biologists contains articles on many of the points above.

Software and code

Policy information about availability of computer code

Data collection	Flow cytometry data was collected with FlowJo v9.9
Data analysis	Analysis of immunologic data was performed using GraphPad Prism 8.4.3 (GraphPad Software).

For manuscripts utilizing custom algorithms or software that are central to the research but not yet described in published literature, software must be made available to editors and reviewers. We strongly encourage code deposition in a community repository (e.g. GitHub). See the Nature Portfolio guidelines for submitting code & software for further information.

Data

Policy information about availability of data

All manuscripts must include a data availability statement. This statement should provide the following information, where applicable:

- Accession codes, unique identifiers, or web links for publicly available datasets
- A description of any restrictions on data availability
- For clinical datasets or third party data, please ensure that the statement adheres to our policy

All data are available in the manuscript and the supplementary material.

Field-specific reporting

Please select the one below that is the best fit for your research. If you are not sure, read the appropriate sections before making your selection.

☒ Life sciences ☐ Behavioural & social sciences ☐ Ecological, evolutionary & environmental sciences

For a reference copy of the document with all sections, see nature.com/documents/nr-reporting-summary-flat.pdf

Life sciences study design

All studies must disclose on these points even when the disclosure is negative.

Sample size	Sample size includes N=47 (N=20 Ad26.COV2.S, N=27 BNT162b2). Based on our previous experience with SARS-CoV-2 vaccine immunogenicity, this sample size provides sufficient power to determine differences in immunogenicity.
Data exclusions	No data were excluded.
Replication	Immunologic measures were performed in duplicate. All technical replicates were successful.
Randomization	Participant demographics were similar (Extended Data Table 1). The study was not randomized.
Blinding	All immunologic assays were performed blinded.

Reporting for specific materials, systems and methods

We require information from authors about some types of materials, experimental systems and methods used in many studies. Here, indicate whether each material, system or method listed is relevant to your study. If you are not sure if a list item applies to your research, read the appropriate section before selecting a response.

Materials & experimental systems

n/a	Involved in the study	
☐	☒	Antibodies
☐	☒	Eukaryotic cell lines
☒	☐	Palaeontology and archaeology
☒	☐	Animals and other organisms
☐	☒	Human research participants
☒	☐	Clinical data
☒	☐	Dual use research of concern

Methods

n/a	Involved in the study	
☒	☐	ChIP-seq
☐	☒	Flow cytometry
☒	☐	MRI-based neuroimaging

Antibodies

Antibodies used	For ELISA and ELISPOT assays anti-macaque IgG HRP (NIH NHP Reagent Program), rabbit polyclonal anti-human IFN-γ (U-Cytech); for ICS assays mAbs from BD against CD279 (clone EH12.1, BB700), CD4 (clone L200, BV711), CD27 (clone M-T271, BUV563), CD8 (clone SK1, BUV805), CD45RA (clone 5H9, APC H7), Ki67 (clone B56, BB515), IL21 (clone 3A3-N2.1, PE), CD69 (clone TP1.55.3, ECD), IL10 (clone JES3-9D7, PE CY7), IL13 (clone JES10-5A2, BV421), IL4 (clone MP4-25D2, BV605), TNF-α (clone Mab11, BV650), IL17 (clone N49-653, BV750), IFN-γ (clone B27; BUV395), IL2 (clone MQ1-17H12, BUV737), IL6 (clone MQ2-13A5, APC), and CD3 (clone SP34.2, Alexa 700).
Validation	mAbs were used according to manufacturer's instructions and previously published methods; mAbs were validated and titrated for specificity prior to use

Eukaryotic cell lines

Policy information about cell lines

Cell line source(s)	HEK293, VeroE6 cells (ATCC)
Authentication	Cell lines were not authenticated.
Mycoplasma contamination	Negative for mycoplasma
Commonly misidentified lines (See ICLAC register)	No commonly misidentified cell lines were used in the study

Human research participants

Policy information about studies involving human research participants

Population characteristics	Participant demographics are reported in Extended Data Table 1.
Recruitment	Participants were recruited from the BIDMC Specimen Biorepository and the COV1001 clinical trial as described in the Methods.
Ethics oversight	BIDMC Institutional Review Board

Note that full information on the approval of the study protocol must also be provided in the manuscript.

Flow Cytometry

Plots

Confirm that:

- [x] The axis labels state the marker and fluorochrome used (e.g. CD4-FITC).
- [x] The axis scales are clearly visible. Include numbers along axes only for bottom left plot of group (a 'group' is an analysis of identical markers).
- [x] All plots are contour plots with outliers or pseudocolor plots.
- [x] A numerical value for number of cells or percentage (with statistics) is provided.

Methodology

Sample preparation	10^6 PBMCs/well were re-suspended in 100 µL of R10 media
Instrument	BD FACSymphony
Software	FlowJo v9.9
Cell population abundance	10^6 PBMC; see Extended Data Figs. 4, 5
Gating strategy	Preliminary FSC/SSC gate and CD3/4/8 gate; see Extended Data Figs. 4, 5

- [x] Tick this box to confirm that a figure exemplifying the gating strategy is provided in the Supplementary Information.
